# Supplementary material for: “Dr. Google, I am in Pain”—Global Internet Searches Associated with Pain: A Retrospective Analysis of Google Trends Data
Source: Int J Environ Res Public Health. 2020 Feb 4;17(3):954. doi: 10.3390/ijerph17030954 (PMC7037174; doi:10.3390/ijerph17030954)
Supplement: Supplementary file 1 [file ijerph-17-00954-s001.pdf]

Supplementary File

Table S1

Checklist for Documentation of Google Trends research.

a) Initial list of pain locations and factors related to pain

Name	Matched as topic related to pain (not disease diagnosis)
Head & Neck	
Headache / Head Pain	Yes, „Headache"
Eye pain	Yes „Eye pain"
Nose pain	No
Ear pain	Yes, „Ear pain"
Toothache	Yes, „Toothache"
Tongue pain	No
Lip pain	No
Sore Throat	Yes, „Sore Throat"
Neck pain	Yes, „Neck pain"
Trunk	
Chest pain / Heart pain	Yes, „Chest pain"
Breast pain	Yes, „Breast pain"
Abdominal pain / Stomache	Yes, „Abdominal pain"
Epigastric pain	Yes, „Epigastric pain"
Umbilical pain	No
Flank pain	Yes, „Abdominal pain"
Hypogastrium pain	No
Groin pain	Yes, „Groin pain"
Back pain	Yes, „Back pain"
Low back pain / Lumbar pain	Yes, „Low back pain"
Pelvic region	
Pelvic pain	Yes, „Pelvic pain"
Penis pain	Yes, „Penile pain"
Testicular pain / Pain of balls	Yes, „Testicular pain"
Rectum pain / Anal pain	Yes, „Rectum pain"
Limbs	
Shoulder pain	Yes, „Shoulder pain"
Clavicle pain	No
Arm pain	No

Forearm pain	No
Wrist pain	Yes, „Wrist pain"
Hand pain / Palm pain	No
Thigh pain	No
Buttock pain	No
Knee pain	Yes, „Knee pain"
Calf pain / Calf cramps	No
Podalgia / Feet pain	Yes, „Podalgia"
Factors	
Dysmennorhea / Painful mennorhea	Yes, „Dysmenorrhea"
Dyspareunia / Sex during intercourse	Yes, „Dyspareunia"
Odynophagia / Pain during swallowing	Yes, „Odynophagia"
Pain during breathing	No
Pain during walking	No

b) Search details

Section/Topic	Checklist item
**Search Variables**	
Access Date	22 July 2019
Time Period	From January 2004 to date of the collection (22 July 2019).
Query Category	All query categories were used
Region	Worldwide
Countries with Low Search Volume	Excluded
**Search Input**	
Non-adjusted	Term recognized as topics: „Abdominal pain", „Back pain", „Breast pain", „Chest pain", „Dysmennorrhea", „Dyspareunia", „Ear pain", „Epigastric pain", „Eye pain", „Groin pain", „Headache", „Knee pain", „Low back pain", „Neck pain", „Odynophagia", „Pelvic pain", „Penile pain", „Podalgia", „Rectal pain", „Shoulder pain", „Sore throat", „Testicular pain", „Toothache", „Wrist pain"
Adjusted	„Abdominal pain" + „Back pain" / „Breast pain" / „Chest pain" / „Dysmennorrhea" / „Dyspareunia" / „Ear pain" / „Epigastric pain" / „Eye pain" / „Groin pain" / „Headache" / „Knee pain" / „Low back pain" / „Neck pain" / „Odynophagia" / „Pelvic pain" / „Penile pain" / „Podalgia" / „Rectal pain" / „Shoulder pain" / „Sore throat" / „Testicular pain" / „Toothache" / „Wrist pain"
**Rationale for Search Strategy**	

For Search Input	The searched topics represents different location and conditions of pain
For Setting Chosen	We chose the abovementioned categories to not limit the output.

Table S2

Use of data generated by Google Trends.

	Interest over time	Region
		Interest by region
Non-adjusted data	Time series analysis: yearly trends, seasonal variation (Table 4, Figure 3)	Countries with the highest RSV of pain-related topics (Table 3)
		Compared breakdown by region
Adjusted data	Proportion of mean RSV to „Abdominal pain" (Table 2, Figure 1)	List of the most common pain-related topics in a specific country (Figures 2A-B, Supplementary Table 2)

RSV – Relative Search Volume

Table S3

The five most common pain-related topics in a specific country.
Relative search volume estimated by using adjusted data of compared breakdown by region (abdominal pain + another pain-related topics).

Country	The five most common pain-related topics (RSV)
Algeria	Abdominal pain (50), Back pain (40), Headache (38), Low back pain (23), Dysmenorrhea (17)
Argentina	Headache (62), Abdominal pain (50), Back pain (41), Sore throat (39), Low back pain (32)
Australia	Headache (54), Abdominal pain (50), Back pain (49), Sore throat (36), Chest pain (26)
Austria	Headache (65), Back pain (52), Sore throat (51), Abdominal pain (50), Toothache (33)
Belarus	Abdominal pain (50), Chest pain (23), Sore throat (23), Back pain (20), Toothache (14)
Belgium	Headache (55), Abdominal pain (50), Back pain (47), Sore throat (32), Low back pain (31)
Bolivia	Headache (53), Abdominal pain (50), Dysmenorrhea (15), Ear pain (13), Odynophagia (3)
Brazil	Headache (53), Abdominal pain (50), Back pain (38), Podalgia (31), Sore throat (26)
Bulgaria	Abdominal pain (50), Headache (43), Sore throat (22), Podalgia (19), Toothache (18)
Canada	Headache (57), Back pain (50), Abdominal pain (50), Sore throat (40), Chest pain (26)
Chile	Headache (57), Abdominal pain (50), Back pain (37), Low back pain (32), Sore throat (23)
China	Abdominal pain (50), Headache (39), Toothache (25), Dysmenorrhea (22), Sore throat (21)
Colombia	Headache (56), Abdominal pain (50), Back pain (30), Low back pain (23), Sore throat (20)
Costa Rica	Headache (52), Abdominal pain (50), Low back pain (23), Ear pain (16), Pelvic pain (4)
Croatia	Headache (53), Abdominal pain (50), Back pain (35), Chest pain (31), Podalgia (28)
Czechia	Abdominal pain (50), Headache (44), Neck pain (41), Back pain (38), Sore throat (35)
Denmark	Headache (67), Abdominal pain (50), Sore throat (44), Back pain (36), Low back pain (32)
Ecuador	Headache (55), Abdominal pain (50), Back pain (28), Low back pain (23), Sore throat (21)
Egypt	Abdominal pain (50), Headache (48), Back pain (30), Low back pain (17), Sore throat (16)
Finland	Headache (58), Abdominal pain (50), Back pain (46), Sore throat (43), Chest pain (30)
France	Abdominal pain (50), Headache (49), Back pain (41), Sore throat (31), Low back pain (26)
Germany	Headache (62), Back pain (54), Abdominal pain (50), Sore throat (48), Chest pain (32)
Greece	Abdominal pain (50), Headache (48), Neck pain (34), Sore throat (22), Back pain (21)
Guatemala	Abdominal pain (50), Dysmenorrhea (13), Ear pain (11), Testicular pain (2), Dyspareunia (1)
Hong Kong	Abdominal pain (50), Headache (46), Sore throat (33), Back pain (25), Low back pain (22)
Hungary	Headache (58), Abdominal pain (50), Sore throat (38), Toothache (33), Back pain (27)
India	Headache (52), Abdominal pain (50), Back pain (46), Podalgia (27), Chest pain (24)
Indonesia	Headache (56), Abdominal pain (50), Toothache (41), Low back pain (21), Chest pain (18)
Iran	Headache (52), Abdominal pain (50), Low back pain (37), Back pain (36), Sore throat (32)
Iraq	Abdominal pain (50), Back pain (36), Low back pain (20), Podalgia (17), Toothache (15)
Ireland	Back pain (56), Headache (54), Abdominal pain (50), Sore throat (38), Podalgia (29)
Israel	Abdominal pain (50), Headache (38), Back pain (37), Sore throat (19), Podalgia (18)
Italy	Headache (68), Back pain (55), Abdominal pain (50), Chest pain (30), Knee pain (30)
Japan	Headache (54), Abdominal pain (50), Low back pain (40), Dysmenorrhea (20), Back pain (15)
Jordan	Abdominal pain (50), Headache (37), Back pain (31), Low back pain (19), Sore throat (19)
Kazakhstan	Abdominal pain (50), Headache (31), Chest pain (22), Sore throat (21), Toothache (13)
Kenya	Abdominal pain (50), Headache (48), Back pain (43), Chest pain (20), Sore throat (20)
Kuwait	Headache (51), Abdominal pain (50), Back pain (38), Low back pain (21), Dysmenorrhea (13)
Malaysia	Headache (52), Abdominal pain (50), Sore throat (32), Toothache (29), Back pain (26)
Mexico	Headache (55), Abdominal pain (50), Back pain (29), Sore throat (27), Low back pain (20)
Morocco	Abdominal pain (50), Headache (43), Back pain (40), Low back pain (23), Toothache (21)
Netherlands	Headache (51), Abdominal pain (50), Back pain (42), Sore throat (29), Podalgia (20)
New Zealand	Headache (56), Back pain (50), Abdominal pain (50), Sore throat (39), Chest pain (26)
Nigeria	Abdominal pain (50), Headache (45), Back pain (35), Sore throat (26), Chest pain (24)
Norway	Headache (65), Abdominal pain (50), Back pain (35), Sore throat (31), Podalgia (23)
Oman	Abdominal pain (50), Dysmenorrhea (14), Breast pain (10), Back pain (0), Chest pain (0)
Pakistan	Headache (55), Abdominal pain (50), Back pain (49), Podalgia (31), Sore throat (31)
Peru	Headache (59), Abdominal pain (50), Back pain (32), Low back pain (32), Sore throat (23)
Philippines	Headache (50), Abdominal pain (50), Back pain (41), Sore throat (31), Dysmenorrhea (28)
Poland	Abdominal pain (50), Headache (40), Sore throat (24), Back pain (21), Podalgia (21)

Portugal	Headache (51), Abdominal pain (50), Back pain (40), Sore throat (27), Podalgia (26)
Romania	Headache (56), Back pain (51), Abdominal pain (50), Neck pain (42), Podalgia (32)
Russia	Abdominal pain (50), Headache (28), Sore throat (24), Chest pain (23), Back pain (18)
Saudi Arabia	Abdominal pain (50), Headache (44), Back pain (32), Sore throat (19), Low back pain (18)
Singapore	Headache (52), Abdominal pain (50), Back pain (40), Sore throat (39), Chest pain (21)
Slovakia	Headache (50), Abdominal pain (50), Sore throat (33), Back pain (27), Toothache (27)
South Africa	Headache (53), Abdominal pain (50), Back pain (43), Chest pain (23), Sore throat (22)
South Korea	Headache (58), Abdominal pain (50), Back pain (44), Chest pain (31), Dysmenorrhea (29)
Spain	Abdominal pain (50), Headache (49), Back pain (38), Low back pain (34), Sore throat (26)
Sweden	Headache (75), Back pain (67), Sore throat (67), Dysmenorrhea (56), Abdominal pain (50)
Switzerland	Headache (61), Back pain (50), Abdominal pain (50), Sore throat (40), Chest pain (27)
Taiwan	Abdominal pain (50), Headache (46), Sore throat (30), Chest pain (22), Low back pain (21)
Thailand	Abdominal pain (50), Headache (45), Back pain (30), Sore throat (26), Pelvic pain (18)
Turkey	Headache (51), Abdominal pain (50), Toothache (37), Groin pain (36), Back pain (34)
Ukraine	Abdominal pain (50), Headache (34), Sore throat (25), Chest pain (24), Back pain (22)
United Arab Emirates	Abdominal pain (50), Headache (49), Back pain (45), Sore throat (28), Chest pain (21)
United Kingdom	Headache (52), Back pain (51), Abdominal pain (50), Sore throat (32), Chest pain (26)
United States	Headache (59), Back pain (52), Abdominal pain (50), Sore throat (40), Chest pain (28)
Venezuela	Abdominal pain (50), Headache (49), Back pain (24), Low back pain (21), Neck pain (18)
Vietnam	Neck pain (62), Abdominal pain (50), Headache (32), Back pain (17), Podalgia (15)

RSV – Relative Search Volume

Datasets are available in Mendeley repository:

DOI: 10.17632/9x6jdhbz5b.1

Kamiński, Mikołaj; Łoniewski, Igor; Marlicz, Wojciech (2020), ""Dr. Google, I am in pain"—Global Internet searches associated with pain: A retrospective analysis of Google Trends data", Mendeley Data, V1, doi:
